# Effects of Alginate Concentration and Ovarian Cells on *In Vitro* Development of Mouse Preantral Follicles: A Factorial Study 

**DOI:** 10.22074/ijfs.2020.5746

**Published:** 2019-11-11

**Authors:** Parisa Jamalzaei, Mojtaba Rezazadeh Valojerdi, Leila Montazeri, Hossein Baharvand

**Affiliations:** 1 Department of Anatomy, Faculty of Medical Sciences, Tarbiat Modares University, Tehran, Iran; 2 Department of Embryology, Reproductive Biomedicine Research Center, Royan Institute for Reproductive Biomedicine, ACECR, Tehran, Iran; 3 Department of Cell Engineering, Cell Science Research Center, Royan Institute for Stem Cell Biology and Technology, ACECR, Tehran, Iran; 4 Department of Stem Cells and Developmental Biology, Cell Science Research Center, Royan Institute for Stem Cell Biology and Technology, ACECR, Tehran, Iran; 5 Department of Developmental Biology, University of Science and Culture, Tehran, Iran

**Keywords:** Alginate, Hydrogel, Ovarian Cells, Preantral Follicle, Tissue Engineering

## Abstract

**Background:**

In the present study, the effects of alginate (ALG) concentration and ovarian cells (OCs) on the devel- opment and function of follicles were simultaneously evaluated.

**Materials and Methods:**

In the first step of this experimental study, preantral follicles were isolated from the ovaries of 2-week-old mice, encapsulated in the absence or presence of OCs in 0.5, 0.75 and 1% ALG hydrogels, and cultured for 14 days. The morphology, diameter, survival and antrum formation rates of the follicles and the maturation of the oocytes were evaluated during culture. In the second step, preantral follicles were cultured in the best chosen ALG concentration, in both the absence and presence of OCs. Following these steps, the amount of DNA fragmentation, the expression levels of connexin 37 and connexin 43 proteins, the secretion levels of estradiol, progesterone and andros- tenedione by the follicles and the quality of mature (MII) oocytes were assessed.

**Results:**

Our data revealed that in the absence of OCs, follicles of 0.5% group showed a higher survival rate than the 0.75 and 1% groups (71.87 vs. 52.52 and 40%, respectively, P<0.05). Nonetheless, the antrum formation rate of the 1% group was higher and its oocyte degeneration rate was lower than that in the other groups. Furthermore, it was observed that co-culture of follicles with OCs relatively increased the follicle diameter, survival, antrum formation, and germinal vesicle (GV) to GV break down (GVBD)/MII transition rates. At last, the comparison of 0.5%-OCs and 0.5%+OCs groups indicated that the co-culture condition resulted in more progesterone production (1.8 ± 0.2 vs. 3.2 ± 0.4 ng/ml, respectively, P<0.05) and also decreased oocytes’ cortical granule abnormalities (100 vs. 40% for 0.5%- OCs and 0.5%+OCs groups, respectively).

**Conclusion:**

The present study revealed that 0.5% ALG hydrogel is relatively suitable for preantral follicle culture, and in the presence of OCs, it mimics the natural ovarian condition better than the higher concentrations of ALG hydrogel.

## Introduction

Isolation and *in vitro* culture of immature ovarian follicles is widely used as a research tool to study the folliculogenesis process (1). It is also a potential alternative to preserve fertility in patients with cancer, who do not have enough time to undergo gonadotropin stimulation before chemotherapy treatments (2).

In general, follicles are cultured in attachment and non-attachment systems. In an attachment culture system, the follicle architecture and the communications of follicular cells are disrupted because of the attachment of granulosa cells to the culture dish, as the mentioned phenomenon might negatively affect the growth and development of the follicles, especially in large mammals (3-5). Therefore, the non-attachment culture system is developed as an alternative way for follicle culture. In the non-attachment culture system, due to the use of a 3D natural or synthetic matrix, the natural structure of the follicles and gap junctions between the follicular cells are preserved well (3, 4). So, this system may be more successful than an attachment system, especially when applied to larger species such as domestic animals and primates (6, 7).

In recent years, alginate (ALG) hydrogel has been largely used in biomedical and tissue engineering applications (8). ALG is a naturally-derived polysaccharide, which is produced by seaweed. It is ionically cross-linked with divalent cations such as calcium (Ca^2+^) to form a gel with a mesh-like structure (9). ALG has been also used for the culture of isolated follicles and has yielded desirable results with follicles from different species (4, 5). However, previous studies have suggested that ALG physical characteristics, which are adjusted by its composition and concentration, could influence the follicles’ survival rate, antrum formation, diameter, maturation, genes expression and hormonal secretions in a species- and stage-specific manner (5, 10-12). 

Moreover, it is shown that the molecular support provided by different cell types could affect follicle growth and development as well as the physical mechanics of the matrix (13, 14). In this regard, recent studies have shown that ovarian cells (OCs) have a stimulatory effect on growth and development of follicles, *in vitro* (15, 16). The OCs could potentially be applied in follicle culture systems in forms of a feeder layer below the encapsulated follicles or they may be co-encapsulated with follicles inside the matrix (15-17). However, it seems that co-encapsulation of OCs with follicles is more practical for follicle culture, as it mimics *in vivo* follicular microenvironment and allows for paracrine signaling as well as the attachments and interactions of granulosa-OCs (17).

Although in many studies the impacts of different ALG concentrations and the rule of OCs on follicle development have been investigated, there is no study that evaluates these two parameters simultaneously to find the best ALG concentration for the culture of both preantral follicles and OCs. Hence, in the first step of the present factorial study, the morphology, diameter, survival, antrum formation and maturation of preantral follicles encapsulated and cultured in different concentrations of ALG hydrogel, in the absence or presence of co-encapsulated OCs (OCs and +OCs-respectively), are evaluated. Then, in the second step, to understand the effects of OCs on the quality of cultured follicles and their oocytes, the preantral follicles were cultured in the best concentration of ALG in the absence or presence of OCs, and were investigated in terms of the amounts of DNA fragmentation in the follicular cells. Since gap junction proteins play a significant role in the folliculogenesis process via transferring nutrients, ions and some nucleotides among follicular cells and oocyte, the changes in their expression might somehow affect follicle development (18, 19). Therefore, the expression of connexin 37 (Cx37) and connexin 43 (Cx43), which are two main gap junction proteins in the follicle structure (20, 21), were also assessed in this study. Finally, the function of the follicles and the quality of the obtained metaphase II (MII) oocytes were examined by evaluating hormonal secretions, cortical granules and spindle/ chromosome abnormality rates.

## Materials and Methods

### Study design

In the first step of this experimental study, preantral follicles were isolated from mice ovaries in five independent replicates, randomly allocated to encapsulate in 0.5, 0.75 and 1% ALG hydrogels in the absence or presence of OCs, and cultured for 13 days. The diameter and morphological appearance of developing follicles were analyzed on days 1, 6 and 13 of culture. Additionally, on day 13, the survival rate of the follicles was calculated and healthy follicles were evaluated with regard to their antrum formation rate. Then, antral follicles were induced by 2.25 IU/ml human chorionic gonadotropin (hCG, Choriomon, Switzerland) and 20-22 hours later, on day 14 of culture, the developmental stages of the obtained oocytes were assessed. After determining the best concentration of ALG based on the larger diameter, higher survival, antrum formation, and maturation rates, in the second step of the study this concentration was used for culturing preantral follicles in the absence or presence of OCs. On day 13 of culture, antral follicles were fixed for investigation of DNA fragmentation and assessment of Cx37 and Cx43 protein levels. Conditioned media from the follicle cultures were collected in three replicates for the measurement of estradiol, progesterone, and androstenedione secretions. Finally, after hCG induction, MII oocytes were collected and evaluated in terms of their cortical granule distribution, meiotic spindle organization, and chromosomal alignment.

### Animals

Female NMRI mice (Pasteur Institute, Iran) were housed in the animal facility of Royan Institute under standard housing conditions, with controlled temperature (20-25°C) and lighting (12 hours light: 12 hours dark). They were handled pursuant to the ethical guidelines set by Royan Institute (ethical permission number: IR.ACECR.ROYAN.REC.1395.93). 

### Isolation and culture of ovarian cells 

Twenty three-four-week-old immature mice were sacrificed by cervical displacement, and their ovaries were isolated in an aseptic condition and placed in ice-cold base medium containing Dulbecco's Modified Eagle’s medium (DMEM, Gibco, UK), penicillin (Gibco, UK), streptomycin sulfate (Gibco, UK), sodium bicarbonate (NaHCO3, Sigma, USA) and 10% fetal bovine serum (FBS, Gibco, UK). Next, the ovaries were cleaned of the bursa and adipose tissue under a stereomicroscope (SZ61, Olympus, Japan). Oocytes and granulosa cells were removed from the ovaries by puncturing follicles with two 29G insulin syringes and then discarded. The remnants were chopped and incubated for 45 minutes at 37°C in 200 µl per ovary of collagenase solution containing 4 mg/ml collagenase IV (Gibco, UK) in serum-free base medium. During this time, the ovarian tissue pieces were pipetted up and down at least 20 times every 10-15 minutes to mechanically disrupt them. To stop the enzymatic activity an equivalent volume of base medium was added to the samples. The isolated cell solution was then filtered through a sterilized 40 µm filter mesh (Falcon, Mexico) and centrifuged at 1800 rpm for 5 minutes. The obtained cells were washed and the final pellet was re-suspended in a known volume of base medium. The cells were transferred to T25 culture flasks containing 4 ml of base medium supplemented with 1% insulin-transferrin-selenium (ITS, Gibco, UK), 1% L-glutamine (Sigma, USA), 1% non-essential amino acids (Gibco, UK) and 0.1% β-mercaptoethanol (Sigma, USA), and then incubated at 37°C in a water-saturated atmosphere of 95% air and 5% CO2 until they reach full confluency. Next, OCs were trypsinized and washed and the viable cells were counted with a trypan blue staining and a Neubauer chamber. Afterward, 1ml aliquots of the cells (5×10<sup>5</sup> cells/ml) were stored in 10% DMSO (Sigma, USA)/FBS at -80°C for later use.

### Isolation of follicles

A total of thirty 12-14-day-old mice were sacrificed by cervical displacement. Mouse ovaries were mechanically dissected under a stereomicroscope at 37°C, using two 29G needles attached to 1ml insulin syringes, and placed in alpha minimum essential medium (α-MEM, Gibco, UK) supplemented with penicillin, streptomycin, NaHCO_3_ , and 10% FBS. Only intact preantral follicles with 2-3 layers of granulosa cells and 100-130 µm in diameter were chosen and divided randomly into experimental groups. 

### Preparation of hydrogels

To make a 1.0% (w/v) ALG solution, 10 mg/ml alginic acid sodium salt (Sigma, USA), 25 mM 4-(2-hydroxyethyl)- 1-piperazineethanesulfonic acid (HEPES, Sigma, USA) and 150 mM sodium chloride (NaCl, Sigma, USA) were dissolved in deionized water, filtered through a sterilized 0.22 µm filter (Millipore, USA) (22), then diluted with sterile 1X phosphate buffered saline (PBS, Takara, Japan) without calcium and magnesium to reach the final concentrations of 0.75 and 0.5% (w/v). In order to prepare hydrogels, cross-linking solution (50 mM calcium chloride (CaCl_2_, Sigma, USA)/140 mM NaCl in deionized water) was mixed with hydrogel solutions.

### Encapsulation and culture

In the first step of the study, groups of 109.83 ± 7.59 preantral follicles were individually encapsulated in 0.5, 0.75 and 1% ALG solutions and in the absence or presence of OCs, in five independent replicates. For cell encapsulation, about 5×103 OCs per follicle were mixed with hydrogel solutions and pipetted in 5-µl droplets on sterile ultra-low attachment culture dishes (Dow Corning, USA). The concentration of OCs in each droplet was determined based on the best results obtained in our pilot study. Afterwards, follicles were individually placed in the 5-µl droplets, cross-linking solution was gently pipetted on top of each droplet, and then incubated at 37°C for 2 minutes. After incubation, the beads were washed with α-MEM medium and then placed into 96-well plates (TPP, Switzerland). Each well contained one bead in 100 µl culture medium [α-MEM supplemented with 5% FBS, 1% ITS, 10 mIU/ml follicle stimulating hormone (FSH, Merck, Germany)]. Lastly, plates were incubated in a 5% CO_2_ incubator at 37°C for 13 days and 50 µl of the medium was replenished every 3-4 days. 

### Assessment of follicle diameters, survival and antrum formation rates

Morphological features and the diameters of developing follicles were assessed on days 1, 6, 13 of culture. The diameters were determined as the mean of two perpendicular measurements of each follicle using ImageJ software (U.S. National Institutes of Health) (23). Moreover, on day 13 of culture, the survival rate of the cultured follicles and antrum formation rate of the survived follicles were evaluated observationally based on the morphological appearances of the follicles and their oocytes. In this regards, extrusion of the oocytes, their dark appearance and surrounding granulosa cells were considered the indications of degeneration. Also, antrum formation was determined as an observable transparent cavity within the granulosa cell masses. 

### Determination of oocytes meiotic maturation

*In vitro* maturation and ovulation of antral follicles were induced by 2.25 IU/ml hCG, on day 13 of culture. For evaluation of meiotic maturation of the oocytes, at 20-22 hours after induction the extruded cumulus-oocyte complexes (COCs) were removed from the culture wells and denuded by gentle pipetting, then the number of germinal vesicle (GV), GV breakdown/metaphase II (GVBD/MII), and degenerated oocytes were determined. 

### Histological processing

In the second step of the study, some preantral follicles were encapsulated in the ultimate concentration of ALG hydrogel from the first step, either in the absence or presence of OCs, and were cultured similar to the first step. On day 13 of culture, survived antral follicles were fixed in 4% paraformaldehyde overnight at 4°C; then the follicles were rinsed twice in PBS, dehydrated in increasing concentrations of ethanol and embedded in paraffin. Next, 5 µm thick slices were prepared and mounted on adhesion slides for DNA and protein analyses. Three sections per group, taken from the middle of three random follicles, were selected for either DNA fragmentation or Cx37 and Cx43 protein expression detection. To prepare the sections, they were first deparaffinized at 60°C for 30-40 minutes, washed in xylene solution for 20 minutes, and rehydrated by rinsing in serially diluted ethanol and water bath.

### Detection of DNA fragmentation

Strand breaks of DNA in apoptotic cells were detected by terminal deoxynucleotidyl transferase-mediated dUTP nick end labeling (TUNEL) assay utilizing the In Situ Cell Death Detection Kit, TMR Red (Roche Diagnostics GmbH, Mannheim, Germany). The procedure was done according to the manufacturer's protocol and previous descriptions (24). In brief, after deparaffinization and rehydration, sections were pretreated with freshly prepared permeabilization solution [0.1% Triton X-100, 0.1% sodium citrate (Sigma, USA)] for 8 minutes at room temperature. After rinsing with PBS/0.05% Tween 20 (PBS-T, Sigma, USA), sections were incubated in TUNEL reaction mixture containing 50 µl enzyme solution (terminal deoxynucleotidyl transferase) and 450 µl label solution (nucleotide mixture in reaction buffer) at 37°C in a humid incubator for 1 hour. Finally, the sections were washed in PBS-T, counterstained in 40,6-diamino-2-phenylindole (DAPI, Sigma, USA), and examined under an inverted fluorescence microscope (Eclipse 50i; Nikon, Japan). All images were processed for publication by the Adobe Photoshop software (CS5.1, Adobe Systems Inc., San Jose, USA).

Red fluorescence was visualized in TUNEL-positive cells. Sections from mouse ovarian tissue were incubated with DNase I recombinant [5 U/ml in 50 mM Tris- HCl, 1mg/ ml bovine serum albumin (BSA), pH=7.5] and were used as a positive control. Negative control sections were incubated with the label solution rather than TUNEL reaction mixture.

To quantify the DNA fragmented follicular cells, all cells found in the sections, either for TUNEL staining or DAPI counterstaining, were counted using ImageJ cell counter plugin; then, the percentage of TUNEL-positive cells was computed.

### Assessment of Cx37 and Cx43 protein expression

After deparaffinization and rehydration, antigen retrieval was performed by incubating the sections of the antral follicles in 0.01 M sodium citrate buffer (pH=6.0, Sigma, USA) for 1 hour in a 96°C oven. The sections were washed two times in PBS-T, and incubated in 10% goat and donkey serums (Sigma, USA) diluted in PBS for 1 hour at 37°C to block non-specific protein bindings in Cx37 and Cx43 immunostainings, respectively. After two PBS washes, the sections were incubated overnight at 4°C with primary antibodies against Cx37 [Primary rabbit polyclonal antibody (ab181701, Abcam, UK)] and Cx43 [primary mouse monoclonal antibody (C8092, Sigma, USA)]. Both primary antibodies were diluted 1:400 in related blocking solutions. Then, the sections were rinsed with PBS carefully and incubated with secondary antibodies [(goat anti-rabbit IgG (H+L) crossadsorbed (Alexa Fluor 488, A11034, Thermo Fisher Scientific, USA) and donkey anti-mouse IgG H&L (Alexa Fluor 488, ab150105, Abcam, UK)] for 1 hour at 37°C. Both secondary antibodies were diluted 1:1000 in related blocking solutions. Lastly, the sections were washed, counterstained with DAPI for 1 minute, and inspected under an inverted fluorescence microscope (Eclipse 50i, Nikon, Japan). Sections from rat heart tissue were used as a positive control. For the negative control, ovarian sections were processed without the primary antibodies.

### Measurement of hormonal secretions

On day 13 of culture, the level of estradiol (E2), progesterone (P4) and androstenedione (A4) hormones were measured in conditioned media collected from 30 cultured antral follicles per group, in three replicates. The hormonal measurement was conducted using mouse ELISA kits (Bioassay Technology Laboratory, China) and according to the kits’ instruction. Data were adjusted for every follicle by dividing each of the measured hormonal secretions by the number of the follicles. According to the kits’ datasheets, the sensitivity of the assay for E2, P4 and A4 were 1.51 ng/L, 0.28 ng/ml and 0.022 ng/ml, respectively. 

### Evaluation of cortical granule distribution, meiotic spindle organization, and chromosomal alignment

Following the assessment of DNA fragmentation and protein expressions, cultured antral follicles were induced by hCG as was explained in detail in the determination of oocytes meiotic maturation section, and then a total number of 20 MII oocytes (10 oocytes per group) were collected in three replicates. Also, 10 *in vivo* matured MII oocytes were gathered as the control group. To get in vivodeveloped oocytes, three 6-8-week-old NMRI mice were injected intraperitoneally by 7.5 IU of pregnant mare’s serum gonadotropin (PMSG, Sigma, USA) followed 48 hous later by 7.5 IU hCG. After 18 hours, the mice were sacrificed, the COCs were isolated from their oviduct ampulla and then denuded. Afterwards, by using pronase (0.5 mg/ml in PBS, Sigma, USA) the zona pellucida of in vivo- and *in vitro*-developed oocytes were removed at 37°C and then oocytes were fixed in 4% paraformaldehyde for at least 1 hour. After washing in PBS with 0.01% Tween 20, oocytes were permeabilized in PBS containing 0.3% BSA (Gibco, UK) and 0.1% Triton X-100 (Sigma, USA) for 15 minutes at room temperature and blocked in PBS containing 0.3% BSA for 1 hour at 37°C. To stain the meiosis spindle and cortical granules, oocytes were incubated in the blocking solution containing anti-alpha tubulin antibody-microtubule marker (FITC) (1:100; Abcam, UK) and rhodamine-labeled Lens Culinaris Agglutinin (LCA) (1:500, Vector Laboratories, Burlingame, CA, USA) for 1 hour at 37°C. Finally, the stained oocytes were washed in PBS-T, counterstained with Hoechst 33342 (1 mg/ml in 1X PBS, Sigma, USA) for 5 minutes at 37°C and mounted on adhesion slides. Fluorescence labeling was detected using an inverted fluorescence microscope (Eclipse 50i, Nikon, Japan) and images were processed by Adobe Photoshop software. The lack of a cortical distribution was considered as the sign of cortical granule abnormality, and disorganized spindle or misaligned chromosomes were considered as an indicator of spindle abnormality.

### Statistical analysis

Statistical differences in follicle survival rates, antrum formation rates, oocyte maturation rates, oocyte abnormality rates and DNA fragmentation were analyzed using GENMOD procedure including function link logit in the model. GENMOD procedure produced odds ratio (OR) as the strength of difference between the groups. Data associated with follicle diameters were analyzed by MIXED procedure including RANDOM and REPEATED statements in the model to identify between and within covariances, respectively. Data pertaining to hormonal secretions were analyzed using GLM procedure. In addition, LSMEANS statement was included in the model to perform multiple comparisons. All analyses were conducted in SAS version 9.4 (SAS Institute Inc., NC, USA). Differences were considered significant at P<0.05. 

## Results

### The first step: determining the best concentration of alginate hydrogel

**Fig 1:**
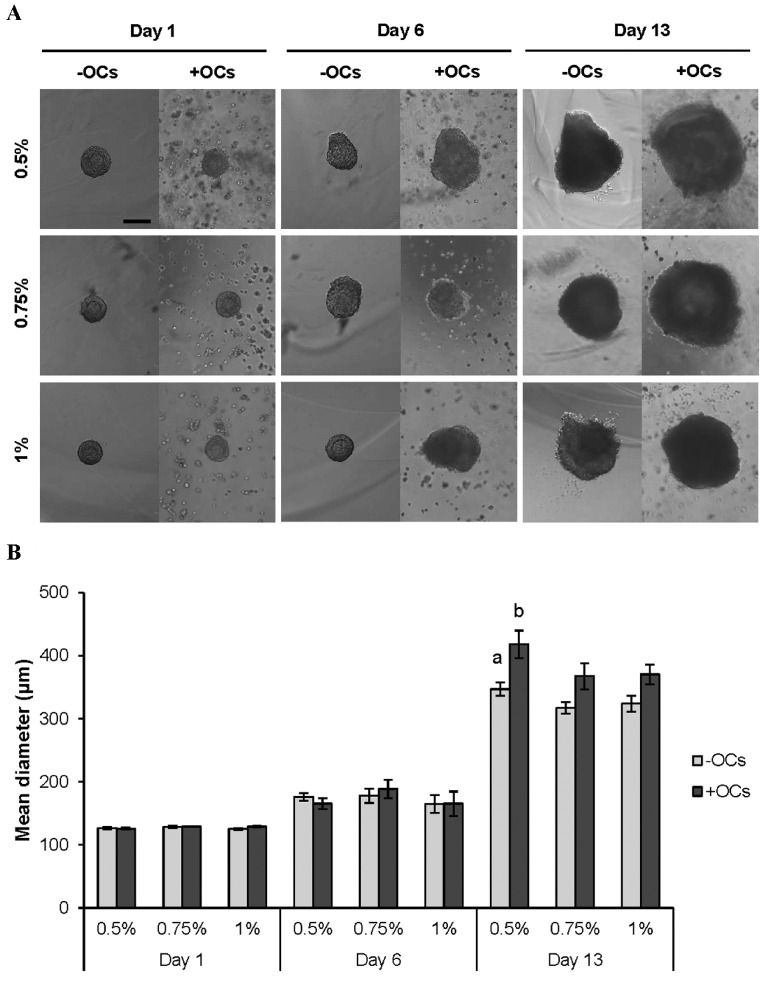
Growth of preantral follicles encapsulated and cultured in 0.5, 0.75 and 1% alginate hydrogels in the absence or presence of ovarian cells (OCs and +OCs-respectively). A. Morphological changes and B. Diameter of the survived follicles on days 1, 6 and 13 of culture. Data are presented as the mean diameter ± standard error. Data points a and b differ significantly (P<0.05, scale bar: 100 µm).

Assessment of the morphologies and diameters of the follicles on days 1, 6 and 13 of culture indicated no significant difference in follicles encapsulated in different concentrations of ALG, either in the absence or presence of OCs. On the other hand, follicles which were co-cultured with OCs had a more spherical shape and relatively larger diameters than the non-co-cultured ones. On day 13 of culture, for instance, the difference in the diameters of 0.5%-OCs and 0.5%+OCs groups was significant (347.18 ± 10.63 vs. 418.14 ± 21.89 µm, respectively, P<0.05, Fig .1A, B).

Assessment of follicle survival rates on day 13 of culture indicated that there was a linear trend towards a better survival rate with lowering ALG concentration, in both the absence and presence of the OCs. However, the difference between 0.5%- OCs group and both 0.75%-OCs and 1%-OCs groups reached statistical significance (71.87 vs. 52.52 and 40%, respectively, P<0.05, Table 1). Also, the comparison of -OCs and +OCs groups revealed that adding the OCs to all hydrogel beads had an affirmative effect on the follicle survival rate, and the difference between 1%-OCs and 1%+OCs groups was significant (40 vs. 63.91%, respectively, P<0.05, Table 1). 

Surprisingly, in the absence of OCs, the proportion of the follicles developed to antral stage was higher in 1% group as compared to the 0.75 % group (75 vs. 59.61%, respectively, P<0.05); while in the presence of OCs there was no significant difference between the groups. On the other hand, all -OCs groups had a relatively lower antrum formation rate than the +OCs ones. Nevertheless, the difference reached statistical difference in 0.5 and 0.75% groups, only (69.59 vs. 88.57% and 59.61 vs. 77.65% for-OCs and +OCs groups, respectively, P<0.05, Table 1).

The evaluation of oocytes obtained from 0.5, 0.75 and 1% ALG-cultured antral follicles showed that there was no significant difference between groups regarding the rates of GV and GVBD/MII oocytes, either in the absence or presence of OCs. However, 0.5% ± OCs groups had a higher rate of degenerated oocytes than the 1% ± OCs ones (P<0.05). Also, it was clear that the oocytes of 0.5%+OCs and 0.75%+OCs groups were more likely to break down their GVs and develop to GVBD or MII stages as compared to the -OCs groups (P<0.05, Table 1).

### The second step: evaluation of the quality and function of cultured follicles

**Fig 2:**
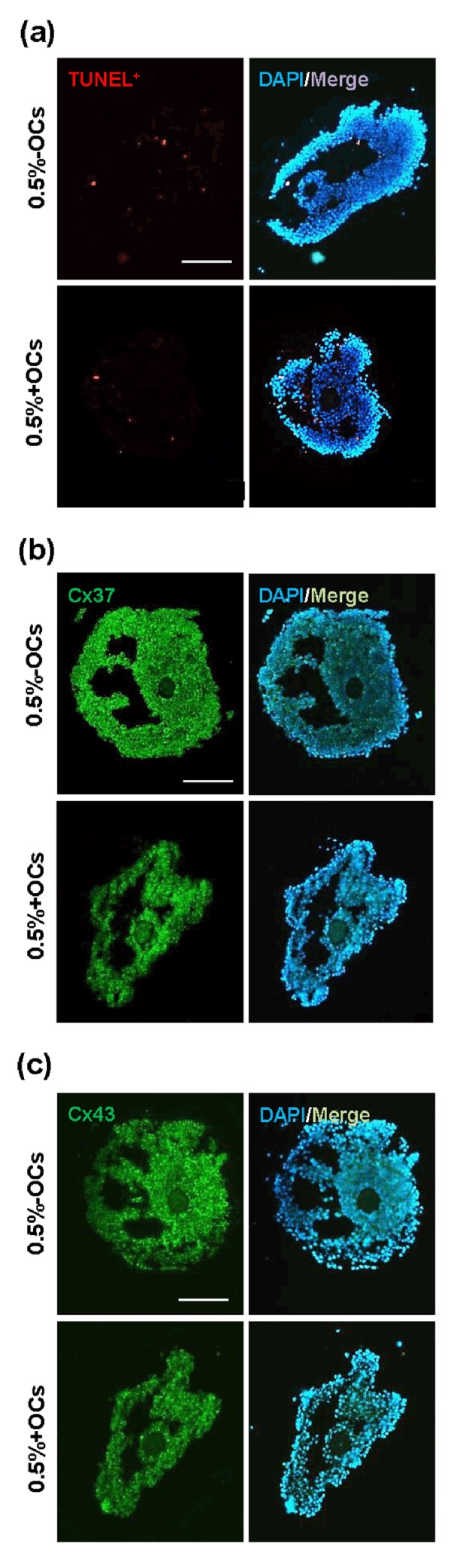
Quality assessment of antral follicles encapsulated and cultured in 0.5% alginate hydrogel in the absence or presence of ovarian cells (OCs and +OCs-respectively), on day 13 of culture. A. TUNEL staining to detect DNA fragmentation in follicular cells. TUNEL-positive cells are stained in red and nuclei in blue (DAPI), B, and C. Immunofluorescence staining to label connexin 37 (Cx37) and connexin 43 (Cx43) proteins; both Cx37 and Cx43 proteins are stained in green and nuclei in blue with DAPI (scale bars: 100 µm).

Based on the first step results, 0.5% ALG hydrogel is potentially best suited for preantral follicle culture, either in the absence or presence of the OCs. In the second step, the evaluation of DNA fragmentation in 0.5% ALG ± OCcultured antral follicles revealed that although a negligible percentage of the follicular cells was TUNEL-positive in both groups, follicles that were co-cultured with OCs demonstrated a relatively lower percentage of DNA fragmentation compared to the non-co-cultured ones (2.2 ± 0.7 vs. 3.9 ± 0.7%, respectively, Fig .2A).

Immunofluorescence staining for Cx37 and Cx43 are displayed in Figure 2B and C as shown, the strong immuno-labeling of Cx37 and Cx43 were observed on granulosa cells of both -OCs and +OCs groups, while qualitatively, no remarkable difference was observed between the two groups.

**Fig 3:**
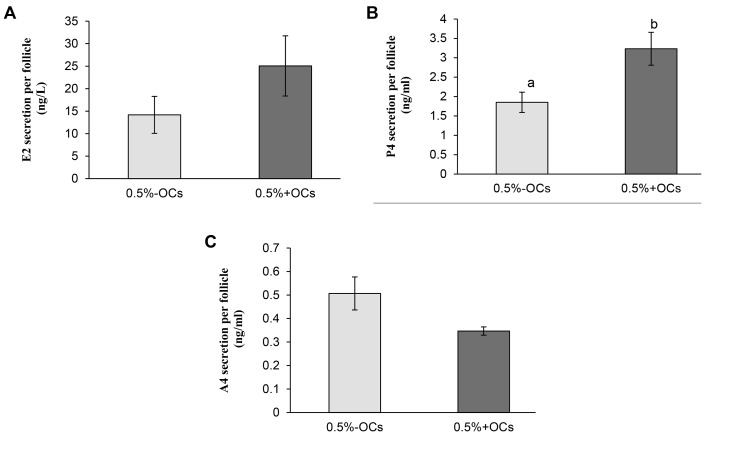
Secretion of hormones by antral follicles encapsulated and cultured in 0.5% alginate hydrogel in the absence or presence of ovarian cells (OCs and +OCs-respectively). A. Estradiol (E2), B. Progesterone (P4), and C. Androstenedione (A4). Conditioned media were collected on day 13 of culture. Data are presented as mean ± standard error. Data points a and b are significantly different (P<0.05).

Hormonal secretion data indicated that there was no significant difference between the groups in the levels of E2 and A4 hormones; however, the level of P4 in the +OCs group was significantly higher than that in the -OCs group (3.2 ± 0.4 vs. 1.8 ± 0.2 ng/ml, respectively, P<0.05, Fig .3A-C). 

**Table 1:** Development of preantral follicles cultured in 0.5, 0.75 and 1% alginate hydrogels in the absence and presence of OCs for 14 days


Groups	Survival rate		Antrum formation rate		Oocyte maturation						
					GV		GVBD/MII			Degenerated	
	-OCs	+OCs	-OCs	+OCs	-OCs	+OCs	-OCs		+OCs	-OCs	+OCs

0.5%	69/96 (71.87)^A^	105/129 (81.39)	48/69 (69.59)^a^	93/105 (88.57)^b^	14/48 (29.16)^a^	8/93 (8.60)^b^	28/48 (58.33)^a^	69/93 (74.19)^b^		6/48 (12.5)^A^	16/93 (17.20)^A^
0.75%	52/99 (52.52)^B^	94/138 (68.11)	31/52 (59.61)^A^^a^	73/94 (77.65)^b^	12/31 (38.70)^a^	5/73 (6.84)^b^	17/31 (54.83)^a^	63/73 (86.30)^b^		2/31 (6.45)	5/73 (6.84)
1%	40/100 (40)^B^^a^	62/97 (63.91)^b^	30/40 (75)^B^	55/62 (88.70)	6/31 (19.35)	9/55 (16.36)	24/31 (77.41)	44/55 (80)		1/31 (3.22)^B^	2/55 (3.63)^B^


OCs; Culture in the absence of ovarian cells, +OCs; Culture in the presence of ovarian cells, GV; Germinal vesicle, GVBD; Germinal vesicle breakdown, and MII; Metaphase II. Data are presented as n (%). A vs. B in each column and a vs. b in each row differ significantly (P<0.05).

**Fig 4:**
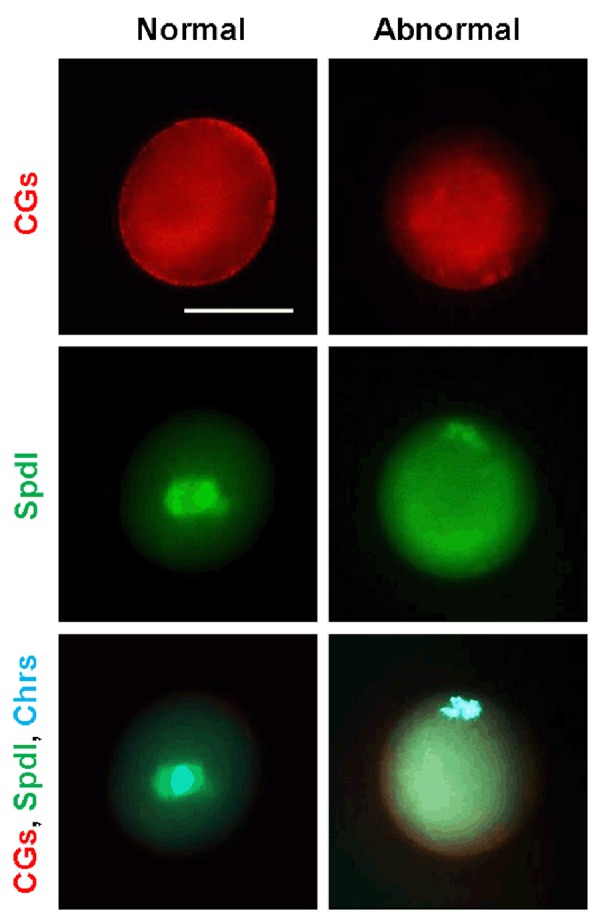
Immunofluorescence staining and abnormality assessment of corti- cal granules (CGs; red), meiotic spindle (Spdl; green) and chromosomes (Chrs; blue) in MII oocytes. Oocytes with cortical distributed cortical gran- ules, a well-organized spindle, and centrally aligned chromosomes were considered as normal oocytes (scale bar: 50 µm).

The normal and abnormal MII oocytes in terms of cortical granule distribution, meiotic spindle organization, and chromosomal alignment are shown in Figure 4. Our data revealed that all in vivo-developed oocytes had a normal cortical granule distribution; whereas 100 and 40% of the oocytes that were developed in 0.5%-OCs and 0.5%+OCs groups, respectively, were abnormal (lack of a cortical distribution of cortical granules) (P<0.05). Concerning meiotic spindle organization, under both in vivo and in vitro conditions, oocytes with abnormal spindle or chromosomal alignments were observed (disorganized spindle or misaligned chromosomes were considered as an indicator of spindle abnormality) (in vivo: 20%; 0.5%-OCs: 40%; 0.5%+OCs: 50%). Interestingly, the presence of OCs had no significant effect on the rate of abnormalities.

## Discussion

The aim of the present study was to simultaneously evaluate the effects of ALG concentration and OCs on the development and function of follicles.

To understand whether the proposed culture condition is ideal for follicle development, the morphological characteristics, diameter, survival and antrum formation rates of the cultured follicles and meiotic resumption of their oocytes were assessed. Previous studies had shown that the rigidity of the matrix used for encapsulation and culture of follicles changes all the above parameters (10, 12, 25). In the studies that have tested mouse ovarian follicle encapsulation and culture in 0.125 to 3% ALG concentrations, it is shown that lower ALG concentrations are more favorable for mouse folliculogenesis (10-12, 25). 

In our study, in the absence of OCs, the survival rate of the follicles cultured with 0.5% ALG was significantly higher than that with 0.75 and 1% ALG. The results were generally consistent with the study of Park et al. (25), which evaluated 0.125 and 0.25% concentrations of ALG hydrogel, where the lower ALG concentration resulted in a higher follicle survival rate. However, some other studies that have examined different ALG concentrations in the range of 0.25 to 3% have reported that ALG rigidity could not affect the follicle survival rate (10-12). It has been previously suggested that increasing the ALG concentration could possibly limit follicles’ access to hormones such as FSH and other nutrients (26, 27). Also, it may increase the mechanical stress exerted on granulosa/ theca cells around the exterior of the follicles, which subsequently influence the oocytes maturation via activating mechano-responsive pathways (28). Since in the present study, oocyte maturation was not significantly altered by increasing the ALG concentration, it could be proposed that the lower survival rate of 1% ALG-cultured follicles was likely due to the small size of the hydrogel’s pores, which limits accessibility of follicular cells to the nutrients, leading to follicle degeneration. Surprisingly, the antrum formation rate in the hydrogel with the most rigidity (1% ALG), was higher than the other ones, while its oocytes degeneration rate was lower. Since it has been confirmed that increasing the matrix rigidity negatively influences the oocyte maturation (10-12), these results were unexpected. Therefore, further investigations are necessary to explain the reasons for these ironic results. 

Interestingly, in the presence of OCs, we observed no remarkable difference between 0.5, 0.75 and 1% ALGcultured follicles in terms of diameter, survival and antrum formation rates and oocyte maturation. Nonetheless, similar to the cultures without OCs, the rate of oocyte degeneration was lower in the group with 1% ALG. However, the comparison of -OCs and +OCs groups showed that the follicles in the +OCs groups had a more spherical shape, a relatively larger diameter, higher survival rate, better antrum formation, and higher GV to GVBD/MII transition rates. The applied OCs in this study comprised of a heterogeneous population of theca/interstitial cells, endothelial cells of the blood vessels, immune cells such as macrophages and smooth muscle cells, which produce high levels of androgens, growth factors and cytokines (16, 29-31). Presumably, these secreted factors affect the follicles via activating signaling pathways involve in both development and growth of the follicles (16, 32).

The cultured antral follicles in 0.5% ALG hydrogel (the best-suited hydrogel for follicle growth and development), in the absence or presence of OCs, also were evaluated for DNA fragmentation, Cx37 and Cx43 protein expressions, hormonal secretions and the quality of their oocytes. Data showed that only a small percentage of the follicular cells were TUNEL-positive after 13 days of culture, either in the absence or presence of OCs. Since the percentage of the TUNEL-positive cells was less than 10%, according to the classification explained in the previous studies (33, 34), both evaluated groups are categorized as minimally damaged, showing an appropriate culture condition, which leads to the high survival rate of the follicular cells.

On the other hand, there was strong immunolabeling of both Cx37 and Cx43, which are two important gap junction proteins in the follicles, in both the absence and presence of OCs. Cx37 and Cx43 are responsible for transportation of nutrients and growth factors essential for the growth and development of the follicles (18-21). Therefore, their higher expression may provide further support for better follicle growth and development. Nonetheless, this increase in Cx37 and Cx43 expressions did not prevent oocyte damage.

Furthermore, the evaluation of hormonal secretion by 0.5% ALG-cultured antral follicles showed that the follicles that were co-cultured with OCs secreted more P4 than the non-co-cultured ones. Earlier studies have found that macrophages enhance progesterone production in the granulosa cells of follicles (35, 36). Therefore, it could be suggested that the higher progesterone secretion by antral follicles co-cultured with OCs might be due to the stimulatory effects of cytokines secreted by the macrophages present in the OCs population. 

Finally, to assess the cytoplasmic and nuclear maturations of 0.5% ALG-developed oocytes, the distribution pattern of cortical granules, the formation of the meiotic spindle and the alignment of chromosomes were evaluated and compared with the in vivo-developed ones. As reported in previous studies, in a normal mature oocyte, cortical granules represent a uniform cortical distribution and the meiotic spindle is also well-assembled. Moreover, a normal mature oocyte contains the correct number and set of chromosomes (37, 38). We have shown that unlike the in vivo-developed oocytes, most of 0.5% ALG -developed oocytes did not show a uniform cortical distribution of cortical granules, neither in the absence nor presence of OCs. This observation is likely due to the clumping of cortical granules within the oocyte cytoplasm as reported in the study by Mainigi et al. (39). So, it could be proposed that an *in vitro* culture of follicles could increase the sensitivity of their oocytes to errors in cortical granule distribution during development. Surprisingly, the oocytes that were co-cultured with OCs, showed a lower percentage of cortical granule abnormalities compared to the non-cocultured ones. This could represent an affirmative effect of the secretions of OCs on factors involved in the clumping of cortical granules, such as oocytes’ Ca2+ concentration or the expression of proteins, which have important roles in fusion of cortical granules (39, 40). Regarding the assessment of spindle and chromosomal abnormalities, in contrast to the results of Mainigi et al. (39), the in vivo and *in vitro* oocytes showed no significant difference. Therefore, it is assumed that our *in vitro* culture systems did not negatively affect spindle formation and chromosomal alignment. However, further evaluations are required to confirm the mentioned assumption.

## Conclusion

In the present study, we showed that both rigidity and concentration of ALG hydrogel influenced the survival rate of the follicles. Indeed, there was a linear trend toward a better survival rate with the lower ALG concentration, in either absence or presence of OCs. Nonetheless, the concentration of ALG did not significantly affect the diameter, antrum formation and maturation rate of the follicles. However, it could be concluded that among 0.5, 0.75 and 1% ALG, the hydrogel with lower concentration is the most suitable for the mouse preantral follicle culture. Moreover, it seems that OCs positively influence follicle diameter, survival, antrum formation and maturation rate, and hormonal secretions. Hence, OCs could be successfully applied to the follicle culture systems in order to improve their culture conditions. 
